# Non-nutritive sweetened beverages versus water after a 52-week weight management programme: a randomised controlled trial

**DOI:** 10.1038/s41366-023-01393-3

**Published:** 2023-10-05

**Authors:** Joanne A. Harrold, Scott Hill, Cristina Radu, Paul Thomas, Paula Thorp, Charlotte A. Hardman, Paul Christiansen, Jason C. G. Halford

**Affiliations:** 1 Department of Psychology, University of Liverpool, Liverpool, UK; 2 School of Psychology, University of Leeds, Leeds, UK

**Keywords:** Obesity, Endocrine system and metabolic diseases

## Abstract

**Background/objective:**

Sugar-sweetened beverages are a substantial source of dietary sugar that can contribute to weight gain and the risk of type 2 diabetes. Dietary guidelines recommend non-nutritive sweetened (NNS) beverages to reduce sugar consumption, however, there is a need for long-term randomised controlled trials on their use. We aimed to compare the effects of NNS beverages and water on body weight during weight loss and maintenance in a behavioural weight management programme.

**Methods:**

In this parallel-group, open-label, controlled equivalence trial, adults with a BMI of 27–35 kg/m^2^ who regularly consumed cold beverages were randomised 1:1 to water or NNS beverages. Participants underwent a group behavioural weight management programme comprising weekly (during the 12-week weight-loss phase) then monthly (during the 40-week weight-maintenance phase) meetings. The primary endpoint was weight change at week 52 (equivalence: two-sided *P* > 0.05). Secondary endpoints included changes in anthropometrics, cardiometabolic risk factors, appetite and activity levels.

**Results:**

Of 493 participants randomised (water: *n* = 246; NNS beverages: *n* = 247), 24.1% were NNS-naïve. At week 52, water and NNS beverages were non-equivalent, with significantly greater weight loss in the NNS beverages group. Participants consuming water maintained a weight loss of 6.1 kg over 52 weeks versus 7.5 kg with NNS beverages (difference [90% CI]: 1.4 kg [–2.6, –0.2]; *p* < 0.05).

**Conclusions:**

During a 52-week behavioural weight management programme, water and NNS beverages were non-equivalent, with weight loss maintained to a statistically greater extent with NNS beverages compared with water. However, this difference was not clinically significant.

**Clinical trial registration:**

This trial is registered with ClinicalTrials.gov: NCT02591134

## Introduction

It is well established that sugar-sweetened beverages are a major source of added sugar in the diet which, when consumed habitually or to excess, can contribute to weight gain and a greater risk of developing type 2 diabetes, cardiovascular disease and certain cancers [1]. Therefore, dietary guidelines recommend lower-calorie options such as water or non-nutritive sweetened (NNS) beverages to reduce overall sugar consumption [2, 3]. However, using NNS beverages as part of a long-term weight management strategy remains a much-debated topic, with controversary relating to their potential effects [3–6]. While some long-term observational studies have reported a positive association between NNS beverage consumption and gains in body weight and body mass index (BMI) [7, 8], meta-analyses and systematic reviews of randomised controlled trials have reported reduced overall energy intake and modest weight loss, with beneficial effects on cardiometabolic health, in participants consuming NNS beverages when compared mostly to sugar-sweetened beverages [9–11]. However, many of the trials included in these reviews were of short- or medium-term duration, and there is still a paucity of data from randomised trials comparing the effects of NNS beverages with water on longer-term weight maintenance following weight loss [12]. Additional long-term randomised controlled trials on this topic will help strengthen the evidence base for making policy recommendations for the use of NNS beverages in weight management programmes.

A previous 52-week randomised controlled trial by Peters and colleagues at the University of Colorado and Temple University compared the effects of NNS beverages and water on weight loss and maintenance [13, 14]. Participants in their trial took part in 12 weeks of active weight loss (using a weekly behavioural weight management programme), followed by 40 weeks of assisted weight maintenance (using monthly lifestyle intervention sessions). The Colorado/Temple trial found that NNS beverages were superior to water for weight loss (–6.2 vs. –2.5 kg, respectively) and for helping participants to better maintain their weight loss throughout the weight-maintenance phase at week 52 [14]. The effectS of non-nutritive sWeetened beverages on appetITe during aCtive weigHt loss (SWITCH) trial expanded on this by using a similar design but with an additional voluntary 52-week extension (after the 40-week assisted weight-maintenance phase) to investigate the effects during unassisted weight maintenance, as well as the inclusion of both NNS beverage-naïve and non-naïve participants [15, 16]. At week 12 in the SWITCH trial, after the active weight-loss phase of the behavioural weight management programme, weight loss was equivalent for participants consuming either NNS beverages or water [16]. Here, we report the effect of water and NNS beverages on body weight at week 52 after completion of both the 12-week active weight loss and 40-week weight-maintenance phases of the SWITCH trial.

A plain language text summary of this article, and accompanying shareable infographic, are available in the Supplemental Material.

## Methods

### Population

The full eligibility criteria for the SWITCH trial have been reported previously [16]. Briefly, healthy adults aged 18–65 years with a BMI of 27–35 kg/m^2^ who regularly consumed >3 cold beverages per week (water, or <2 l per day of NNS or sugar-sweetened beverage) from within a 50-mile radius of the county of Merseyside, England (of which Liverpool is the principal city) were included. Habitual beverage consumption was assessed using a screening questionnaire that asked participants to list the cold beverages consumed in the previous week, with answers counted by the investigators. Exclusion criteria included drinking <3 chilled beverages per week, recent/current smokers, specific health conditions (i.e., diabetes, gastrointestinal or cardiovascular disease), food allergies, excessive alcohol intake, taking medication/supplements known to affect weight, regular intense exercise, dieting or significant weight loss, or bariatric surgery before screening.

### Trial design

SWITCH is a parallel-group, open-label, randomised controlled trial conducted in three phases (12-week active weight loss, 40-week assisted weight maintenance and a voluntary 52-week non-assisted maintenance extension phase), conducted at the University of Liverpool, England. Written informed consent was given by all participants. Remuneration for trial participation consisted of £300 for participants who completed the first 52 weeks, £100 for those who also completed the voluntary 52-week extension, and a maximum of £330 for those who took part in the additional assessments of appetite probe days (data not reported here; £130) and dual-energy X-ray absorptiometry (DXA; £200).

Full details on protocol amendments and approval, including ethical approval, have been reported elsewhere [15, 16].

### Interventions

Participants were randomised to NNS beverages or water using a computer-generated sequence in blocks of 4 and 6 to ensure equal numbers in both groups (1:1). Randomisation was stratified by sex (male/female), age (18–35, 36–50 and 51–65 years), BMI (<30 and ≥30 kg/m^2^) and NNS naïveté (categorised as naïve or non-naïve if NNS beverages constituted 0– ≤ 25% and >26–100%, respectively, of beverage choices in the 5 years before screening, assessed as part of the screening questionnaire on habitual consumption) to ensure a balance of characteristics between groups [16].

Participants were asked to consume at least two servings (each 330 ml) per day of intention-to-treat NNS beverages or water, which could be still or carbonated. For participants who drank the minimum number of cold beverages to be eligible for the trial (three per week), this would represent a complete replacement of their usual consumption. For participants who drank the maximum amount of NNS or sugar-sweetened beverages to be eligible (2 l per day) and were randomised to the NNS group, this would mean replacing at least two daily servings of these particular drinks with their assigned trial beverages; if randomised to the water group, these participants were asked to abstain from all NNS beverages (including adding sweeteners to hot beverages). While all participants were permitted to consume sugar-sweetened beverages, they were provided with nutrition education on healthy dietary patterns, including how to reduce the number of calories consumed (such as by limiting these beverages). Participants in the NNS group could also consume water. For NNS beverages, a range of 20 different branded options were available, with each 330 ml serving containing ≤20.9 kJ (5.0 kcal) per 8 oz/≤ 8.8 kJ (2.1 kcal) per 100 ml. For water, at least two daily servings were to be bottled water, with additional tap water as needed. Trial beverages (two 330 ml servings per day) were provided by the investigators, funded by the sponsor. Adherence was assessed through daily online beverage logs, returning empty packaging (where possible during the coronavirus disease 2019 [COVID-19] pandemic), completing an online Food Frequency Questionnaire for consumption during the previous month at baseline and regular intervals throughout the trial (monthly during week 0–12, quarterly during week 12–52), and completing 3-day food diaries at baseline, week 12 and week 52.

In line with the 12-week weight-loss phase of this trial [16], the 40-week weight-maintenance phase was also based on the cognitive-behavioral interventions used in the Colorado/Temple trial [13, 14, 17]. After the initial weight-loss phase, during which participants attended weekly behavioural weight-loss sessions, participants switched to monthly group sessions held by the same qualified nutritionist, with additional support as needed, whilst they continued to consume their assigned beverages [15]. Participants completed exercise and diet diaries to facilitate self-monitoring and to allow group leaders to provide appropriate feedback. Group sessions covered key themes building on weight loss to focus on maintenance (e.g., the ‘energy gap’, weight plateaus, emotional and situational eating, and the role of exercise and physical activity) and were supplemented with supportive resources and monthly weigh-ins. Participants could miss up to three of the monthly group sessions before they were excluded from the trial on the grounds of non-compliance with the protocol.

### Outcomes and assessments

The primary endpoint of the trial was the change in body weight (kg) from baseline to week 52 (assisted weight-maintenance phase post weight loss; reported here). A voluntary *non-assisted* maintenance phase post *assisted* weight-maintenance phase also measured change in body weight up to week 104; this is to be reported separately.

Secondary endpoints included changes from baseline to week 52 in: waist and hip circumference; glycaemic control; fasting lipids; liver function; hunger (on a 0–100 mm visual analogue scale ranging from ‘not at all hungry’ to ‘extremely hungry’); sugar and sweetener consumption (using the Sugar and Sweetener Food Frequency Questionnaire [SSFFQ], higher scores indicating higher consumption of estimated added sugar or sweeteners in the previous month [15]); and activity level (number of steps assessed using an activity tracker [Fitbit Charge HR®]). Change from baseline to week 52 in body composition was assessed in a subset of participants using full-body DXA scans.

Body weight was measured at baseline and monthly, as were waist and hip circumference, hunger visual analogue scale and SSFFQ. Fasting blood samples and DXA measurement (post-overnight fasting) were taken in a subset of participants. Physical activity was monitored for 1 week at baseline, week 12 and week 52, except during March 2020 (due to the COVID-19 pandemic). The number of steps were hidden to participants, with the device appearing like a normal watch. If data could not be extracted from the devices, it was treated as missing. The number of steps per day were averaged across the week for each participant. If fewer than 50 steps were recorded on any 1 day during the week of the assessment, the average of the remaining days was used instead.

Other deviations to the planned trial protocol in response to England’s COVID-19 restrictions in 2020 and 2021 included: reduced frequency of trial beverage deliveries—with greater quantities of trial beverages per delivery—to minimise social contact; submission of photos by participants of empty packaging to measure adherence; online group sessions conducted via the Zoom platform, which maintained the planned curriculum but with interactive elements; and participant provision of self-reported body weight, waist and hip measurements using a secure online questionnaire, with the same model of electronic scale and tape measure sent to all participants’ homes along with detailed instructions. To assess the potential impact of weight collection location on the primary endpoint, a comparison of self-reported and clinic-collected body-weight measurements was performed. Some individual trial visits were conducted online using a questionnaire (via the Qualtrics® platform), which precluded the collection of blood pressure and blood samples from some participants.

### Statistical analysis

This trial tested the equivalence of NNS beverages to water on weight loss, defined as a two-sided *p* value > 0.05 at week 52. Including an attrition rate of 27%, as reported in the Colorado/Temple trial [14], a sample of 316 participants (*n* = 158 per group; minimum 248 [*n* = 124 per group] excluding attrition) would provide 90% power to detect a ± 1.5-kg weight change difference between groups at week 52 [15].

Endpoints were assessed using an analysis of covariance (ANCOVA), with the blinded trial group as a predictor and baseline value of the outcome of interest (e.g., baseline body weight for predicting week-52 weight) as a covariate. The primary analysis used data from participants who completed the trial up to week 52 (complete cases analysis). The primary analyses were repeated on two data sets in which missing data were imputed via different mechanisms. The first used multiple imputation, with data imputed 50 times through predictive mean matching, and the second used last observation carried forward analysis. Sensitivity analyses were also conducted for the changes in body weight and waist and hip circumference using the same ANCOVA, but with the inclusion of additional covariates (age, sex, location of weight measurement [self- vs. clinic-collected] and NNS beverage naïveté [non-naïve vs. naïve]).

## Results

### Participants

**Fig. 1:**
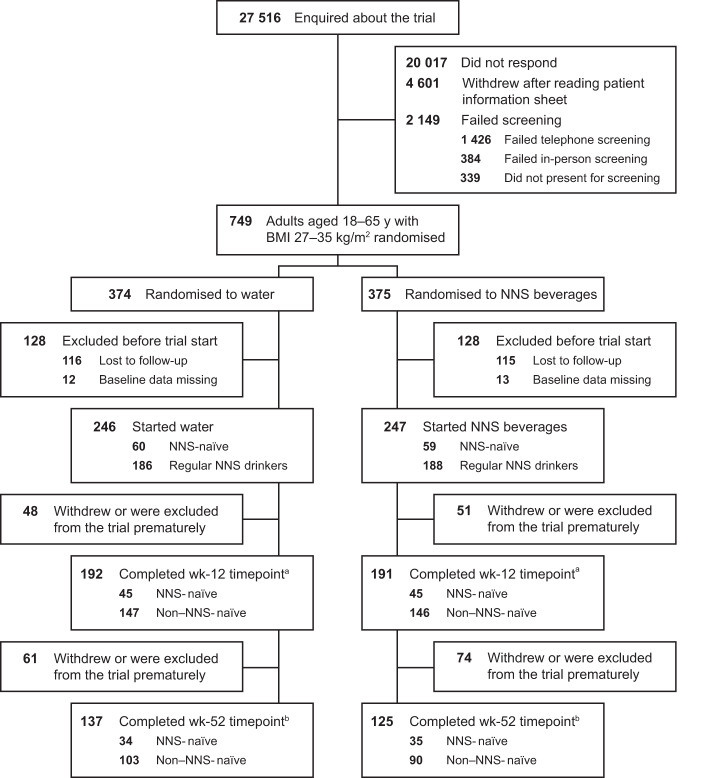
Flow of participants. *NNS* non-nutritive sweetened. Some data have been reproduced with permission from Harrold JA, Hill S, Radu C, Thomas P, Thorp P, Hardman CA, et al. Effects of non-nutritive sweetened beverages versus water after a 12-week weight loss programme: a randomised controlled trial. Obesity (Silver Spring). 2023;31(8):1996–2008. © 2023 The Authors. ^a^Week 12 data were missing for 11 participants (*n* = 6 for water; *n* = 5 for NNS beverages) who remained in the trial. ^b^Week 52 data were missing for four participants (*n* = 2 for water; *n* = 2 for NNS beverages) who remained in the trial. These individuals were, therefore, not included in the analyses using the complete cases data set for the week-52 timepoint but were included in the analyses using the multiple imputation and last observation carried forward data sets. They may also contribute to analyses at future timepoints.

A total of 493 participants were randomised to the trial and initiated treatment (water: *n* = 246; NNS beverages: *n* = 247) between July 2016 and December 2021. The 12-week timepoint was completed by 383 participants [16]. The 52-week timepoint was completed by 262 participants (53.1%; water: *n* = 137; NNS beverages: *n* = 125), who were included in the primary analysis of the complete cases data set (Fig. 1, Supplementary Fig. 1); 135 participants withdrew or were prematurely excluded from the trial and thus had data imputed for the analyses using the two imputed data sets. Of the participants who completed the week-52 timepoint, 93.6% attended the monthly behavioural weight-loss group sessions. Based on beverage logs, compliance with assigned trial beverages (two 330 ml servings per day) at week 52 was high at 98.2% and 98.6% in the NNS and water groups, respectively. Partial compliance (one 330 ml serving per day) was 1.1% and 0.6% and non-compliance (zero servings per day) was 0.7% and 0.8% for the NNS and water groups, respectively.

Baseline characteristics were generally well balanced across the two groups in all randomised participants and among those who completed week 52, with a slight imbalance in mean fasting serum insulin and gamma-glutamyl transferase concentrations (likely due to outliers), as reported previously [16] (Table 1). Overall, most participants were female (70.0%), had a mean (SD) age of 45.4 (11.6) years and BMI of 31.3 (2.3) kg/m^2^; the majority (75.9%) were non-naïve to NNS beverages. Baseline characteristics were generally comparable with the overall population when stratified by week-52 completion status or NNS beverage naïveté, and among those who provided blood samples or were in the DXA subset (Supplementary Tables 1, 2, 3 and 4).

**Table 1:** Baseline characteristics for all randomised participants and week-52 completers.

Variable	Water	NNS beverages
Randomised participants^a^		
Participants, *n*	246	247
Age, year	46.0 ± 11.2	44.7 ± 12.0
Female sex, *n* (%)	165 (67.1)	180 (72.9)
BMI^b^, kg/m^2^	31.3 ± 2.3	31.3 ± 2.2
Body weight, kg	90.4 ± 11.1	89.9 ± 11.2
NNS beverage naïveté^c^		
Non-naïve, *n* (%)	186 (75.6)	188 (76.1)
Naïve, *n* (%)	60 (24.4)	59 (23.9)
Week-52 completers		
Participants, *n*	137	125
Age, year	48.6 ± 10.2	47.0 ± 11.2
Female sex, *n* (%)	92 (67.1)	90 (72.9)
BMI^b^, kg/m^2^	31.2 ± 2.3	31.3 ± 2.3
Body weight, kg	89.6 ± 10.9	89.6 ± 11.5
NNS beverage naïveté^c^		
Non-naïve, *n* (%)	103 (75.2)	90 (72.0)
Naïve, *n* (%)	34 (24.8)	35 (28.0)

There were no significant differences between completers and non-completers in BMI, sex or NNS naïveté (*p* > 0.05 for comparisons). Non-completers were, however, younger than completers (*p* < 0.01). Data are mean ± SD or *n* (%).

*NNS* non-nutritive sweetened.

^a^Data have been reproduced with permission from Harrold JA, Hill S, Radu C, Thomas P, Thorp P, Hardman CA, et al. Effects of non-nutritive sweetened beverages versus water after a 12-week weight loss program: a randomised controlled trial. Obesity (Silver Spring). 2023;31(8):1996–2008. © 2023 The Authors.

^b^BMI was measured at screening as part of trial eligibility assessments.

^c^Naïve was defined as NNS beverages comprising 0– ≤ 25% of drink choices in the 5 years to screening; these individuals could be regular consumers of water or sugar-sweetened beverages. Non-naïve was defined as NNS beverages comprising >26–100% of drink choices in the 5 years to screening.

### Anthropometrics

A total of 262 participants had body-weight measurements at week 52 and contributed to the primary outcome, of whom 114 had clinic-collected data, 93 had self-collected data and 55 had clinic-collected baseline data and self-collected week-52 data.

**Fig. 2:**
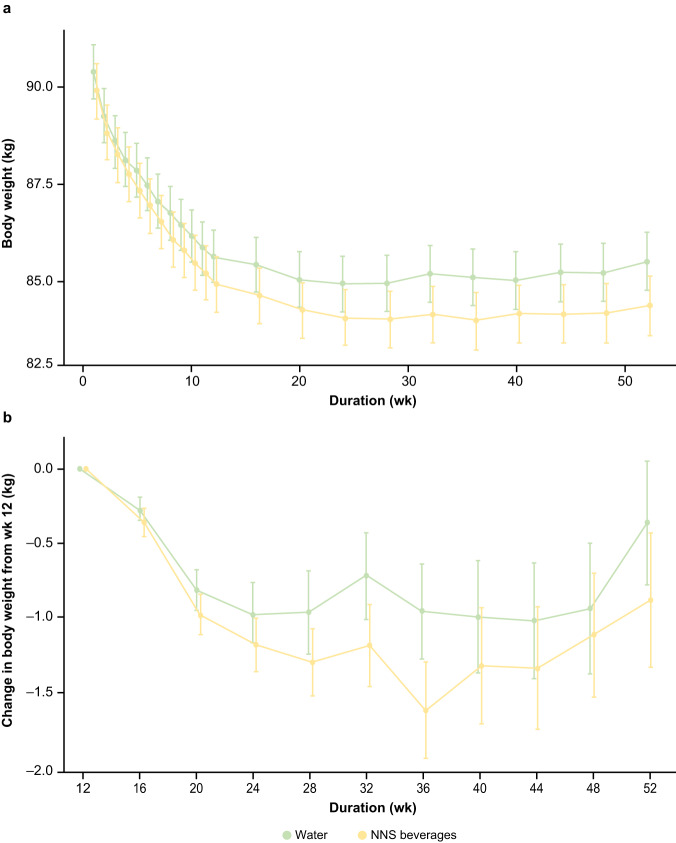
Time profile of weight change over 52 weeks in the complete cases data set. **a** The change from baseline to week 52. **b** The change from week 12 to week 52. Primary analysis of the complete cases data set, which included all participants with data at baseline and week 52. The error bars are the standard error of the mean. The week-16 timepoint represents the first body-weight measurement during the weight-maintenance phase. NNS non-nutritive sweetened.

The greatest rate of weight loss occurred during the first 12 weeks of the trial in both groups (Fig. 2a). Weight loss appeared to be greater with NNS beverages compared with water from the beginning of the trial. Maximum weight loss was reached at week 44 with water and week 36 with NNS beverages (Fig. 2b). Both groups started to regain weight after these timepoints, with a slower rate of increase in the NNS beverages group compared with the water group.

**Fig. 3:**
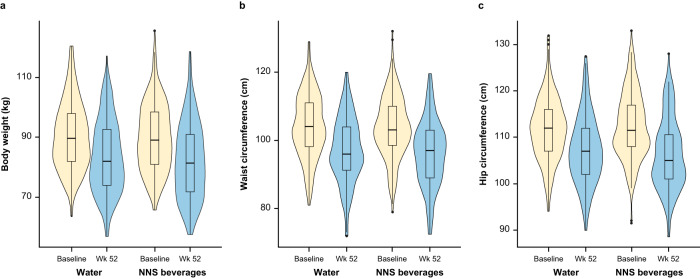
Violin plot showing the effect of trial beverage on selected variables at baseline and week 52 in the complete cases data set. **a** Effect on body weight. **b** Effect on waist circumference. **c** Effect on hip circumference. Primary analysis of the complete cases data set, which included all participants with data at baseline and week 52. The shaded areas refer to the kernel density (the probability that a member of the population will have a given value), the boxes show the interquartile range, the horizontal lines in the centre of the boxes are the median values and the whiskers refer to 1.5× the interquartile range. NNS non-nutritive sweetened.

At week 52, both groups had significant reductions in body weight from baseline (Table 2, Fig. 3a). Mean weight change was –6.1 kg with water versus –7.5 kg with NNS beverages for the primary analysis using the complete cases data set. As can be seen in Table 2, the groups were not equivalent in terms of weight loss as there was a significant difference in the changes from baseline between the groups; the weight loss with NNS beverages was significantly greater than weight loss with water. The final sample of 262 participants at week 52 gave a power marginally larger than that required to detect the 1.5-kg difference (*n* = 248); this meant the 1.4-kg change in weight was non-equivalent. When the primary analysis was repeated using the multiple imputation and last observation carried forward data sets, there were no significant differences in weight loss between the water and NNS beverage groups (Table 2).

**Table 2:** Effects of trial beverage on the primary endpoint at week 52 in the complete cases, multiple imputation and last observation carried forward data sets.

Group	Baseline	Week 52	Change	90% CI for change^a^
Complete cases data set				
Body weight, kg				
Water (*n* = 137)	89.6 ± 10.9	83.5 ± 12.0	−6.1 ± 5.8**	−6.9, −5.3
NNS beverages (*n* = 125)	89.6 ± 11.5	82.1 ± 12.6	−7.5 ± 5.9**	−8.4, −6.6
Between-group difference	0.0 ± 22.5	1.4 ± 24.7	1.4 ± 11.7*	0.2, 2.6
Imputed data set				
Body weight, kg				
Water (*n* = 246)	90.4 ± 11.1	84.1 ± 11.9	−6.3 ± 6.3**	−7.0, −5.6
NNS beverages (*n* = 247)	89.9 ± 11.2	82.7 ± 12.3	−7.1 ± 5.9**	−7.7, −6.5
Between-group difference	0.5 ± 22.3	1.3 ± 24.2	0.8 ± 12.2	−0.1, 1.7
Last observation carried forward data set				
Body weight, kg				
Water (*n* = 246)	90.4 ± 11.1	85.5 ± 11.8	–4.9 ± 5.3**	–5.5, –4.3
NNS beverages (*n* = 247)	89.9 ± 11.2	84.4 ± 12.0	–5.5 ± 5.1**	–6.0, –5.0
Between-group difference	0.5 ± 22.3	1.1 ± 23.7	0.6 ± 10.4	–0.2, 1.4

Primary analysis of the complete cases data set, which included all participants with data at baseline and week 52, the multiple imputation data set, for which missing data were imputed using predictive mean matching (50 imputations), and the last observation carried forward data set, for which missing data were imputed using participants’ last observed value. Data were assessed using linear models comparing mean differences, with between-group differences assessed using an ANCOVA, with the blinded trial group as a predictor and baseline value of the outcome of interest as a covariate. Data are mean ± SD unless otherwise specified.

*ANCOVA* analysis of covariance, *NNS* non-nutritive sweetened.

*p < 0.05.

**p < 0.001.

^a^For the test of equivalence with two-sided *p* value > 0.05.

When assessing the impact of various covariates on weight at week 52 in the primary analysis of the complete cases data set, baseline weight had a significant effect of 0.97 kg (95% CI: 0.9, 1.0; *p* < 0.001). After controlling for this, trial beverage had a significant effect on weight of –1.4 kg [95% CI: –2.9, –0.0]; *p* = 0.049), with those in the NNS beverages group having lower weight than the water group. Body-weight measurements were unaffected by collection location (self- vs. clinic-collected; Supplementary Fig. 2). In sensitivity analyses using the three data sets, baseline weight and NNS beverage assignment (complete cases data and last observation carried forward data), and baseline weight and age (multiple imputation) had significant effects on week-52 weight (Supplementary Tables 5, 6 and 7). The other predictors of sex, weight collection location and NNS naïveté had no effects. When controlling for these covariates, there was a significant difference in week-52 weight between groups in the analysis of complete cases data (effect of beverage group [95% CI]: –1.5 kg [–2.9, –0.1]; *p* = 0.040; Supplementary Table 5). Findings were similar for the last observation carried forward data set but were not significant for the multiple imputation data set (Supplementary Tables 6 and 7).

Waist and hip circumference were also significantly reduced from baseline in both groups (Fig. 3b, c, Table 3). The difference between groups was significant for hip, but not waist, circumference. In primary analyses using the complete cases data set, baseline waist circumference had a significant effect on week-52 waist measurement, while both baseline hip circumference and assigned NNS beverages had a significant effect on week-52 hip measurements (Supplementary Tables 8 and 9). When controlling for covariates in the sensitivity analyses, there was no significant effect of beverage group on week-52 waist circumference, but the effect on hip circumference was maintained. Other covariates had no effects on week-52 waist and hip measurements.

**Table 3:** Effects of trial beverage on the secondary endpoints at week 52 in the complete cases data set.

Group	Baseline	Week 52	Change	95% CI for change
Waist circumference, cm				
Water (*n* = 135)	104.1 ± 8.8	97.1 ± 9.7	−7.0 ± 6.9**	−8.2, −5.8
NNS beverages (*n* = 121)	104.8 ± 8.8	96.2 ± 9.5	−8.6 ± 7.2**	−9.9, −7.3
Between-group difference	−0.8 ± 17.6	0.9 ± 19.2	1.6 ± 14.2	−0.1, 3.3
Hip circumference, cm				
Water (*n* = 135)	111.7 ± 6.4	107.3 ± 7.3	−4.4 ± 5.4**	−5.3, –3.5
NNS beverages (*n* = 120)	112.4 ± 7.0	106.4 ± 7.6	−6.0 ± 5.5**	−7.0, –5.0
Between-group difference	−0.7 ± 13.5	0.9 ± 14.9	1.6 ± 11.0*	0.2, 3.0
Systolic blood pressure, mmHg				
Water (*n* = 111)	136.4 ± 16.4	130.2 ± 15.6	−6.2 ± 17.5**	−9.5, –2.9
NNS beverages (*n* = 105)	135.1 ± 12.5	131.2 ± 14.7	−3.9 ± 14.5*	−6.7, –1.1
Between-group difference	1.3 ± 29.1	–1.0 ± 30.3	−2.4 ± 32.0	−6.7, 1.9
Diastolic blood pressure, mmHg				
Water (*n* = 111)	83.6 ± 9.0	79.1 ± 10.1	–4.5 ± 9.8**	–6.3, –2.7
NNS beverages (*n* = 105)	83.3 ± 8.8	79.5 ± 10.5	–3.8 ± 10.2**	–5.8, –1.8
Between-group difference	0.3 ± 17.8	–0.4 ± 20.6	–0.7 ± 20.0	–3.4, 2.0
Total cholesterol, mmol/l				
Water (*n* = 68)	5.5 ± 1.1	5.2 ± 1.1	–0.3 ± 0.6**	–0.4, –0.2
NNS beverages (*n* = 66)	5.3 ± 0.8	5.2 ± 0.9	–0.1 ± 0.6	–0.2, 0.0
Between-group difference	0.2 ± 1.9	0.0 ± 2.0	–0.2 ± 1.3	–0.4, 0.0
HDL cholesterol, mmol/l				
Water (*n* = 68)	1.5 ± 0.4	1.4 ± 0.3	0.0 ± 0.2	–0.0, 0.0
NNS beverages (*n* = 66)	1.5 ± 0.4	1.6 ± 0.4	0.1 ± 0.2**	0.1, 0.1
Between-group difference	–0.1 ± 0.8	–0.2 ± 0.7	–0.1 ± 0.5**	–0.2, –0.0
LDL cholesterol, mmol/l				
Water (*n* = 68)	3.3 ± 0.9	3.1 ± 0.9	–0.2 ± 0.6*	–0.3, –0.1
NNS beverages (*n* = 66)	3.2 ± 0.8	3.0 ± 0.8	–0.2 ± 0.6*	–0.3, –0.1
Between-group difference	0.2 ± 1.7	0.1 ± 1.7	0.0 ± 1.1	–0.2, 0.2
Non-HDL cholesterol, mmol/l				
Water (*n* = 68)	4.0 ± 1.0	3.7 ± 1.0	–0.3 ± 0.6**	–0.4, –0.2
NNS beverages (*n* = 66)	3.8 ± 0.8	3.5 ± 0.9	–0.3 ± 0.6*	–0.4, –0.2
Between-group difference	0.3 ± 1.9	0.2 ± 1.9	0.0 ± 1.3	–0.2, 0.2
Triglycerides, mmol/l				
Water (*n* = 68)	1.5 ± 0.8	1.4 ± 0.7	–0.2 ± 0.5*	–0.3, –0.1
NNS beverages (*n* = 66)	1.3 ± 0.5	1.1 ± 0.5	–0.2 ± 0.6*	–0.3, –0.1
Between-group difference	0.2 ± 1.3	0.2 ± 1.2	0.0 ± 1.1	–0.2, 0.2
Total cholesterol:triglyceride ratio				
Water (*n* = 68)	4.0 ± 1.2	3.8 ± 1.1	–0.3 ± 0.7*	–0.5, –0.1
NNS beverages (*n* = 66)	3.6 ± 1.0	3.3 ± 0.9	–0.4 ± 0.7**	–0.6, –0.2
Between-group difference	0.4 ± 2.2	0.5 ± 2.0	0.1 ± 1.4	–0.1, 0.3
HbA_1c_, mmol/mol				
Water (*n* = 69)	36.3 ± 3.7	35.7 ± 3.3	–0.7 ± 2.9*	–1.4, –0.0
NNS beverages (*n* = 66)	36.3 ± 3.3	36.4 ± 3.2	0.1 ± 2.3	–0.5, 0.7
Between-group difference	0.0 ± 7.0	–0.8 ± 6.5	0.8 ± 5.2	–0.1, 1.7
Fasting plasma glucose, mmol/l				
Water (*n* = 67)	5.1 ± 0.5	5.0 ± 0.5	–0.1 ± 0.4	–0.2, –0.0
NNS beverages (*n* = 66)	5.0 ± 0.4	5.0 ± 0.5	0.0 ± 0.4	–0.1, 0.1
Between-group difference	0.1 ± 1.0	0.0 ± 0.9	0.0 ± 0.8	–0.1, 0.1
Fasting serum insulin (SI units), pmol/l				
Water (*n* = 63)	87.8 ± 78.1	76.1 ± 53.4	–11.7 ± 77.3	–30.8, 7.4
NNS beverages (*n* = 65)	77.7 ± 43.6	63.6 ± 34.3	–14.1 ± 40.3*	–23.9, 4.3
Between-group difference	10.1 ± 127.0	12.5 ± 90.0	2.4 ± 123.9	–19.1, 23.9
AST, U/L				
Water (*n* = 62)	22.0 ± 5.9	20.5 ± 5.4	–1.5 ± 6.1	–3.0, 0.0
NNS beverages (*n* = 62)	22.2 ± 10.0	20.4 ± 5.4	–1.8 ± 9.4	–4.1, 0.5
Between-group difference	–2.0 ± 16.5	0.1 ± 10.8	0.3 ± 15.9	–2.5, 3.1
ALT, U/L				
Water (*n* = 67)	25.7 ± 15.3	20.7 ± 11.8	−5.0 ± 10.8**	−7.6, –2.4
NNS beverages (*n* = 62)	25.1 ± 19.8	20.3 ± 10.0	−4.9 ± 15.4*	−8.7, –1.1
Between-group difference	0.6 ± 35.6	0.5 ± 21.8	−0.1 ± 26.8	−4.7, 4.5
GGT, U/L				
Water (*n* = 43)	27.3 ± 19.9	23.4 ± 15.1	−3.9 ± 12.2*	−7.5, –0.3
NNS beverages (*n* = 46)	53.5 ± 148.8	41.3 ± 82.8	−10.4 ± 71.1	−30.9, 10.1
Between-group difference	−26.2 ± 208.9	–17.9 ± 117.1	8.3 ± 100.1	−12.5, 29.1
Hunger VAS^a^, mm				
Water (*n* = 133)	43.4 ± 28.0	40.8 ± 25.8	−2.7 ± 34.7	−8.6, 3.2
NNS beverages (*n* = 116)	40.0 ± 28.4	40.0 ± 30.9	0.0 ± 37.1	−6.8, 6.8
Between-group difference	3.5 ± 56.6	0.9 ± 57.5	−2.6 ± 72.2	−11.6, 6.4
Sugar consumption^b^, score points				
Water (*n* = 125)	114.6 ± 43.1	71.4 ± 37.3	−43.2 ± 49.9**	–51.9, –34.5
NNS beverages (*n* = 111)	112.0 ± 46.8	66.3 ± 36.1	−45.7 ± 42.6**	–53.6, –37.8
Between-group difference	2.6 ± 90.3	5.1 ± 73.4	2.6 ± 92.5	–9.2, 14.4
Sweetener consumption^b^, score points				
Water (*n* = 125)	15.7 ± 12.5	2.5 ± 6.0	–13.1 ± 12.8**	–15.3, –10.9
NNS beverages (*n* = 111)	14.5 ± 11.3	15.7 ± 11.9	1.2 ± 8.5	–0.4, 2.8
Between-group difference	1.1 ± 23.8	–13.2 ± 19.2	–14.3 ± 21.5**	–17.0, –11.6
Activity level^c^, steps per day				
Water (*n* = 93)	8 467.3 ± 3 173.9	8 463.7 ± 3 663.3	–3.6 ± 3 706.6	–756.9, 749.7
NNS beverages (*n* = 98)	8 629.0 ± 3 469.3	9 497.9 ± 4 320.6	868.9 ± 3 733.5*	129.7, 1 608.1
Between-group difference	–161.7 ± 6 644.3	–1 034.2 ± 7 996.5	–872.5 ± 7 441.9	–1 928, 182.9
DXA subset				
Fat mass, kg				
Water (*n* = 27)	35.4 ± 4.6	30.3 ± 7.4	–5.1 ± 6.0**	–7.4, –2.8
NNS beverages (*n* = 30)	36.5 ± 5.1	30.4 ± 7.6	–6.1 ± 6.0**	–8.2, –4.0
Between-group difference	–1.1 ± 9.6	–0.1 ± 15.1	1.0 ± 12.0	–2.1, 4.1
Fat-free mass, kg				
Water (*n* = 27)	52.9 ± 10.8	52.0 ± 10.9	–0.8 ± 1.7*	–1.4, –0.2
NNS beverages (*n* = 30)	53.3 ± 10.9	52.3 ± 10.9	–1.0 ± 1.7*	–1.6, –0.4
Between-group difference	–0.5 ± 21.7	–0.3 ± 21.9	0.2 ± 3.4	–0.7, 1.1
Android fat distribution, %				
Water (*n* = 27)	48.6 ± 4.7	43.5 ± 8.8	–5.2 ± 7.3*	–8.0, –2.4
NNS beverages (*n* = 30)	49.5 ± 5.4	43.7 ± 7.7	–5.8 ± 7.2**	–8.4, –3.2
Between-group difference	–0.9 ± 10.1	–0.2 ± 16.6	0.7 ± 14.5	–3.1, 4.5
Gynoid fat distribution, %				
Water (*n* = 27)	42.5 ± 9.2	38.8 ± 9.6	–3.6 ± 4.6**	–5.3, –1.9
NNS beverages (*n* = 30)	42.4 ± 7.4	38.9 ± 8.0	–3.5 ± 3.9**	–4.9, –2.1
Between-group difference	0.0 ± 16.8	–0.1 ± 17.8	–0.1 ± 8.6	–2.3, 2.1

Primary analysis of the complete cases data set, which included all participants with data at baseline and week 52. Data were assessed using an ANCOVA, with the blinded trial group as a predictor and baseline value of the outcome of interest as a covariate. Data are mean ± SD; *n* number of participants with data available. During COVID-19 pandemic restrictions, assessments were not available for all participants (blood samples could not be taken for some participants while others failed to self-report).

*ANCOVA* analysis of covariance, *ALT* alanine aminotransferase, *AST* aspartate aminotransferase, *COVID-19* coronavirus disease 2019, *DXA* dual-energy X-ray absorptiometry, *GGT* gamma-glutamyl transferase, *HbA*_*1c*_ glycated haemoglobin, *HDL* high-density lipoprotein, *LDL* low-density lipoprotein, *NNS* non-nutritive sweetened, *SSFFQ* Sugar and Sweetener Food Frequency Questionnaire, *VAS* visual analogue scale.

*p < 0.05.

**p < 0.001.

^a^Assessed using a 0–100 mm VAS anchored at ‘not at all hungry’ and ‘extremely hungry’.

^b^Assessed using the SSFFQ [15]. The SSFFQ assessed the previous month consumption of sugar or sweetener in foods and drinks based on frequency and portion estimates, with higher scores indicating higher consumption.

^c^The number of steps per day averaged across 1 week.

In the DXA subset, which comprised 57 participants (water: *n* = 27; NNS beverages: *n* = 30), there were significant reductions from baseline in fat mass, fat-free mass and android and gynoid fat distribution from baseline to week 52 in both groups. However, there were no significant differences in body composition endpoints between water and NNS beverages (Table 3).

### Biomarkers

Significant improvements from baseline were observed for most biomarkers at week 52 in both groups (Table 3). There was a significant difference between groups in the changes in high-density lipoprotein cholesterol, driven by a modest increase from baseline in the NNS beverages group (0.0 vs. 0.1 mmol/l). There were no significant differences between groups for the changes in the other biomarkers assessed.

### Hunger and sweetener consumption

There were no significant changes in hunger consumption from baseline in either treatment group (Table 3). Sweetener consumption (caloric or non-caloric) was significantly reduced from baseline in the water group, but not the NNS beverages group (–13.1 vs. +1.2 score points), resulting in a statistically significant difference between them, as expected. Sugar consumption was significantly reduced from baseline to a similar extent in both groups.

### Activity

Activity levels, measured as the average number of steps taken per day over 1 week, decreased with water but increased significantly from baseline with NNS beverages at week 52; however, the difference between the groups was not significant (Table 3).

## Discussion

At week 52, following the active weight loss and assisted weight-maintenance phases of the SWITCH trial, water and NNS beverages were not equivalent in terms of weight loss. Participants in both groups lost weight during the trial (6.1 kg and 7.5 kg with water and NNS beverages, respectively; differences between baseline and week 52 were statistically significant). However, the weight loss in the NNS beverages group was greater than in the water group, a difference that was statistically significant. Consuming NNS beverages also had a significant effect on week-52 weight when baseline body weight and other covariates were controlled for in two of the three sensitivity analyses. Although the difference in weight loss between the groups was statistically significant, it is important to note that this did not reach the 1.5-kg difference identified for clinical significance [15]. The results were statistically significant because the small increase in power compared with the protocol meant that this difference between the groups could be observed. The increase in power was due to increased recruitment to account for the effects of the COVID-19 pandemic and a greater-than-expected dropout rate.

The observed weight loss was accompanied by corresponding improvements from baseline in all other anthropometric measures, most biomarkers and sugar consumption in both groups. The reduction in hip circumference with NNS beverages was significantly greater compared with water and the effect remained significant when both baseline hip circumference and trial beverage group were controlled for in a sensitivity analysis. There was also a significant difference between the groups in the change in high-density lipoprotein cholesterol, driven by a modest increase from baseline in the NNS beverages group compared with no change in the water group. As expected, consumption of sweeteners (either caloric or non-caloric, assessed using the SSFFQ) decreased from baseline in the water group, with no change in the NNS beverages group because of the continued consumption of NNS beverages in this group, resulting in a statistically significant between-group difference.

The results of this analysis build on our previous findings after the initial 12-week weight-loss phase of the trial [16], and suggest that both water and NNS beverages may aid weight loss and subsequent maintenance in people taking part in behavioural weight management programmes, regardless of NNS naïveté. This conclusion is broadly consistent with that of the similar Colorado/Temple group trial [14], upon which SWITCH was based. However, the greater weight loss with NNS beverages compared with water reported in the Colorado/Temple group trial was both statistically and clinically significant [14], whereas in our trial the difference between the groups was statistically, but not clinically, significant. Older trials investigating NNS in food as well as beverages have also found they can assist with maintaining weight loss when used as part of a weight management programme. In one such trial, individuals with obesity lost more weight and maintained their weight loss during a 2-year weight management programme when consuming NNS beverages food and beverages compared with those who did not [18]. In a 6-month trial, substitution of sugar-sweetened beverages with NNS beverages or water resulted in significant reductions in weight, waist circumference and systolic blood pressure in adults with overweight and obesity, with greater weight reductions reported with NNS beverages consumption versus water [19]. In contrast, a recent randomised controlled trial reported greater weight loss with water compared with NNS beverages among regular NNS beverage drinkers over an 18-month weight management programme [20]. However, differences in weight-loss programme, size, timing of beverage consumed (i.e., before, during or after meals) and a baseline study population of NNS beverage drinkers may account for this finding. Another trial reported no significant differences in weight change between participants with central adiposity who consumed sugar-sweetened beverages, NNS beverages or unsweetened beverages, although this could have been due to insufficient power to detect differences [21].

Despite randomised controlled trials often reporting beneficial or neutral effects of NNS beverages on body weight [9, 10, 12, 22, 23], conflicting evidence on their effects comes from lower-quality, observational studies, which typically do not include repeated analysis or substitution [24]. Additionally, recent guidance from the World Health Organization indicated that the majority of randomised controlled trials on this topic lasted 3 months or less [25], highlighting a need for longer-term data. Some review articles summarising observational studies suggest the consumption of NNS beverages can have long-term adverse effects on body weight and related health outcomes, including impaired glucose metabolism and increased risk of comorbidities [10, 26–28]. However, a network analysis of 33 reviews found that most review articles that reported a neutral or beneficial relationship between NNS consumption and body weight cited randomised controlled trials, whereas those that reported a negative relationship typically cited observational studies [11]. A potential explanation for this incongruence is that observational studies (e.g., prospective cohort studies) can mistakenly confuse the direction of causation between NNS use and weight gain or other adverse effects. It may be that the predisposition for weight gain or development of complications occurs first, which can cause individuals to switch to, or increase the use of, NNS beverages (i.e., reverse causation) [10, 29, 30]. Prospective cohort studies also cannot eliminate the potential for residual confounding, despite accounting for the many statistical adjustments made. Indeed, a recent review of prospective cohort studies in over 400 000 adults with varying cardiovascular risk factors (including type 2 diabetes) found NNS beverage consumption was associated with weight reduction and a reduced incidence of obesity, coronary heart disease and all-cause mortality, comparable to that with water when the influence of reverse causality and residual confounding was mitigated [24].

Strengths of the SWITCH trial include the randomised controlled design, 1-year duration and mixed population of NNS-naïve and non-naïve participants. This allows the trial to provide robust, high-quality data on the effects of NNS beverages and water after both a 12-week assisted weight-loss phase [16] and a 40-week assisted maintenance phase. The final phase of this trial will assess long-term effects of NNS beverages over a voluntary 52-week period of unassisted weight maintenance. Both NNS beverage-naïve and non-naïve participants helped to address any effects of prior experience of NNS beverage consumption, increasing confidence in the primary outcome. The proportion of NNS beverage-naïve participants in our trial, both based on the entire starting population and only amongst those who completed the week-52 timepoint, was consistent with the proportion of adults who reported not consuming any NNS-containing food or drink in a UK-based study (~25%) [31]. Because some participants had to take some measurements themselves and self-report during the COVID-19 pandemic, the sensitivity analyses also included the location of weight measurement as a covariate. These analyses and the comparison of clinic- versus self-reported body weight showed that location had no effect on weight at week 52, further increasing confidence.

The main limitation of the trial is the potential lack of generalisability, considering it was conducted at a single site in England and did not collect racial or ethnicity data. This limits consideration of the potential impact of race or ethnicity on our results, as well as their generalisability to wider populations or ethnic groups. A second limitation was the low completion rate of the week-52 timepoint, particularly compared with the similar Colorado/Temple trial (53% vs. 73% of participants who started treatment completed week 52) [14]. Potential reasons for this include the COVID-19 pandemic, which occurred during the SWITCH trial, and the initial 2-year duration of the trial, which participants may have considered too onerous. To address the impact of the pandemic, multiple sensitivity analyses were performed to compare clinic- and self-collected body-weight measurements at baseline and week 52, with missing values imputed. To address the level of commitment required by participants, the second year of the trial was made voluntary.

## Conclusion

After completing a 52-week behavioural weight management programme, which focused on 12 weeks of active weight loss followed by 40 weeks of weight maintenance, the difference in body weight between water and NNS beverages at the end of this phase was non-equivalent. However, although statistically significant, this difference did not reach clinical significance. The final voluntary 52-week period of unassisted weight maintenance in this trial will assess whether the discontinuation of routine nutrition awareness visits will have an impact on further maintaining weight loss or preventing weight gain in both groups.

### Supplementary information

Supplemental material

## Data Availability

Participant data are not publicly available but can be requested from the corresponding author after study completion. Requests should be reasonable and accompanied by research proposals that have received appropriate ethical approval. Data will be made available in an anonymised format in compliance with applicable privacy and data protection laws.
