# Supplementary material for: Non-nutritive sweetened beverages versus water after a 52-week weight management programme: a randomised controlled trial
Source: Int J Obes (Lond). 2023 Oct 5;48(1):83–93. doi: 10.1038/s41366-023-01393-3 (PMC10746539; doi:10.1038/s41366-023-01393-3)
Supplement: Supplementary file 1 — Supplemental material [file 41366_2023_1393_MOESM1_ESM.docx]

SUPPLEMENTAL MATERIAL

**Non-nutritive sweetened beverages versus water after a 52-wk weight management programme: a randomised controlled trial**

Joanne A. Harrold^1^, Scott Hill^1^, Cristina Radu^1^, Paul Thomas^1^, Paula Thorp^1^, Charlotte A. Hardman^1^, Paul Christiansen^1^ and Jason C.G. Halford^1,2^

^1^Department of Psychology, University of Liverpool, Liverpool, UK

^2^School of Psychology, University of Leeds, Leeds, UK

[Plain language summary of publication – text 2](#_Toc141799354)

[Plain language summary of publication – infographic 5](#_Toc141799355)

[Supplemental Table 1 Baseline characteristics for all participants stratified by wk-52 completion. 6](#_Toc141799356)

[Supplemental Table 2 Baseline characteristics for all randomised participants stratified by NNS naïveté^a^. 7](#_Toc141799357)

[Supplemental Table 3 Baseline characteristics for all randomised participants who provided blood samples. 8](#_Toc141799358)

[Supplemental Table 4 Baseline characteristics for all randomised participants who were in the DXA subset. 9](#_Toc141799359)

[Supplemental Table 5 Effects of different covariates on body weight at wk 52 – sensitivity analysis of complete cases data. 10](#_Toc141799360)

[Supplemental Table 6 Effects of different covariates on body weight at wk 52 – primary and sensitivity analyses of multiple imputation data. 11](#_Toc141799361)

[Supplemental Table 7 Effects of different covariates on body weight at wk 52 – primary and sensitivity analyses of last observation carried forward data. 12](#_Toc141799362)

[Supplemental Table 8 Effects of different covariates on waist circumference at wk 52 – primary and sensitivity analyses of complete cases data. 13](#_Toc141799363)

[Supplemental Table 9 Effects of different covariates on hip circumference at wk 52 – primary and sensitivity analyses of complete cases data. 14](#_Toc141799364)

[Supplemental Fig. 1 CONSORT flow diagram 15](#_Toc141799365)

[Supplemental Fig. 2 Comparison of body weight by location of weight measurement. 16](#_Toc141799366)

[Supplemental references 17](#_Toc141799367)

# Plain language summary of publication – text

**Why was the SWITCH study done?**

Often, people who are trying to lose weight, including those with overweight or obesity, drink water or sugar-free, sweetened drinks (which can be labelled ‘Diet’ or ‘Zero’) to lower the amount of sugar in their diet. However, results from different studies disagree on whether sugar-free drinks can affect how much weight people can lose. The SWITCH study was done to provide more information on this topic and to see if there were any differences in body weight between people who drank sugar-free drinks and people who drank water whilst on a weight management programme.

**Who could take part in the SWITCH study?**

To take part, people had to be 18 y old or over, with overweight or obesity and without any major health issues. They also had to regularly drink cold drinks, such as milk, water or carbonated (fizzy) drinks.

**How was the SWITCH study done?**

The SWITCH study was split into three parts. This paper reports the results at the end of part 2 (at wk 52), building on the weight loss results in part 1 (wk 12).^1^

Part 1: active weight loss

Part 1 lasted 12 wk. The results from this part of the study have already been published [1]. During part 1, participants did weekly sessions of a group weight-loss programme and daily exercise. The sessions involved learning about healthy eating and included counselling for food-related behaviour. The goal was to help people learn how to manage their weight more successfully through changes in their lifestyle.

Part 2: assisted weight maintenance

Part 2 lasted 40 wk. At the end of part 2, the participants had been in the study for a total of 52 wk. During this part, people carried on with the group weight management sessions, but they were only held once every mo with monthly weigh-ins. They were also asked to continue exercising every day and learned about long-term changes they could make to help keep weight off over time.

Part 3: unassisted weight maintenance

Part 3 lasts for 1 y. At the end of part 3, participants will have been in the trial for a total of 2 y. The results from this part of the study are not included in this paper as it is currently ongoing. During part 3, people no longer join the group weight management sessions and are instead asked to manage their weight themselves. Participation in part 3 was optional.

**What did the SWITCH study test?**

At the start of the study, the people taking part were split into two equal groups: one group could only drink sugar-free sweetened drinks each day (chilled or at room temperature, not hot drinks) and the other group had to drink water each day (everyone could still drink sugar-sweetened drinks if they wanted to).

The main aim of the SWITCH study was to see how people’s body weight changed during the study. Additional outcomes that are known to be linked to body weight were also measured. These included the size of people’s waist and hips, their blood pressure, how much fat and sugar were in their blood, how well their livers were working and how many steps they were taking. People were also asked broadly how much sugar and sweeteners they were eating and drinking and how hungry they felt.

The results for the people who drank sugar-free drinks were compared with the results for people who drank water.

**What did the SWITCH study find at wk 52?**

Of the 493 people who started the study, 262 stayed in the study until the end of wk 52. Everyone lost weight over the 52 wk. People who drank sugar-free drinks lost slightly more weight than people who drank water. Nearly all other results linked to weight also improved in both groups. There were no differences between the people who drank sugar-free drinks and those who drank water for any of these other changes apart from the size of people’s hips (the size of people’s hips got smaller in both groups, but the people who drank sugar-free drinks had a bigger reduction than the people who drank water) and the level of good cholesterol in their blood (people who drank sugar-free drinks had a slight increase in good cholesterol whereas it stayed the same in people who drank water).

**What does this mean?**

These findings suggest that both sweetened, sugar-free drinks and water are helpful for weight management when taking part in group weight management sessions.

# Plain language summary of publication – infographic


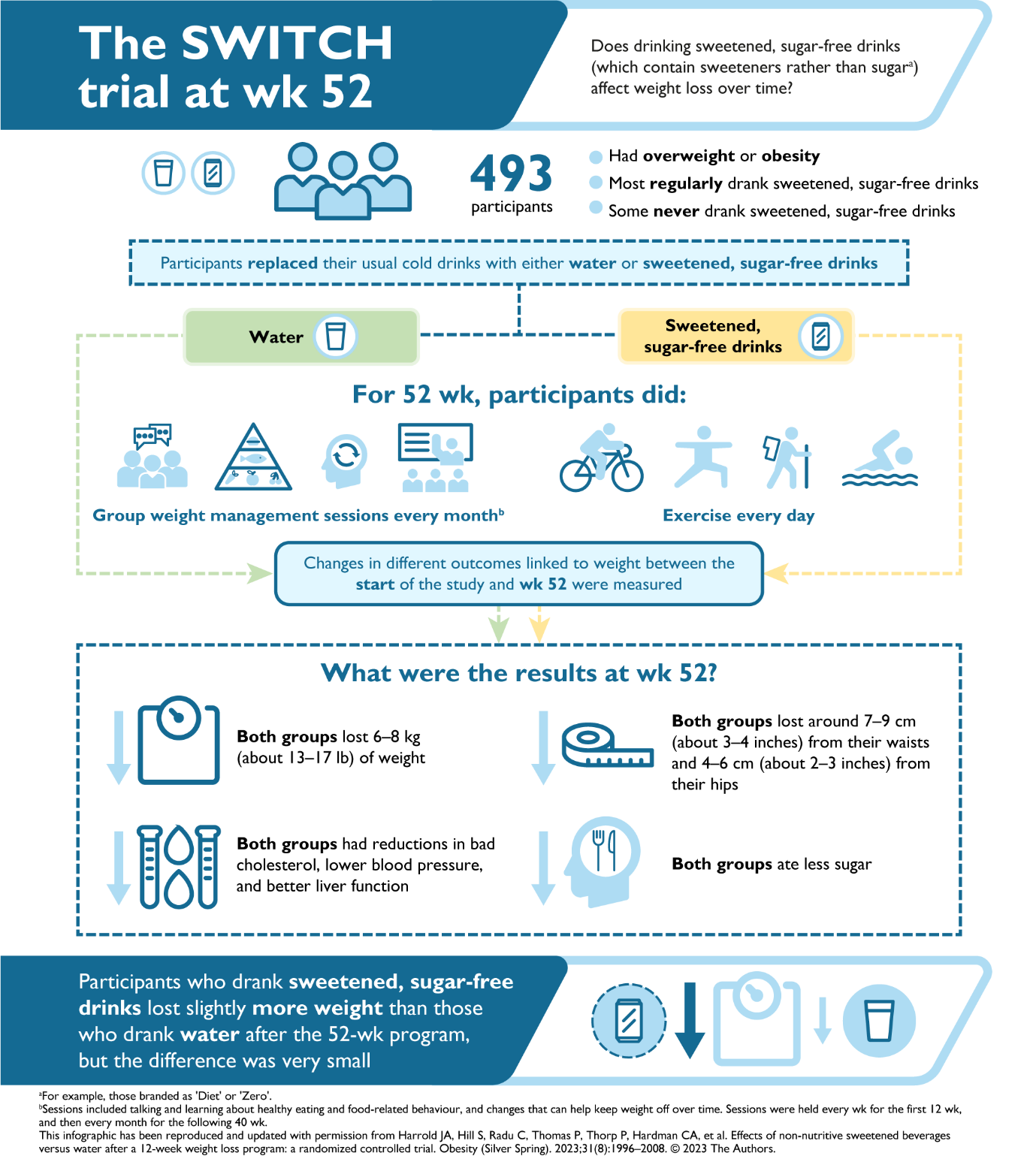


Supplemental Table 1 Baseline characteristics for all participants stratified by wk-52 completion.

| **Variable** | **Completed wk 52 (*n* = 262)** | **Did not complete wk 52 (*n* = 231)^a^** |
| --- | --- | --- |
| Age, y | 47.9 ±10.7 | 42.6 ±12.0 |
| Female sex, *n* (%) | 182 (69.5) | 163 (70.6) |
| BMI^b^, kg/m^2^ | 31.2 ±2.3 | 31.4 ±2.2 |
| Body weight, kg | 89.6 ±11.2 | 90.7 ±11.1 |
| NNS beverage naïveté^c^ |  |  |
| Non-naïve, *n* (%) | 193 (73.7) | 181 (78.4) |
| Naïve, *n* (%) | 69 (26.3) | 50 (21.6) |

*NNS* non-nutritive sweetened.

Data are mean ±SD or *n* (%).

^a^Four participants did not complete wk-52 weight data collection but remained in the trial.

^b^BMI was measured at screening as part of trial eligibility assessments.

^c^Naïve was defined as NNS beverages comprising 0–≤25% of drink choices in the 5 y to screening; these individuals could be regular consumers of water or sugar-sweetened beverages. Non-naïve was defined as NNS beverages comprising >26–100% of drink choices in the 5 y to screening.

Supplemental Table 2 Baseline characteristics for all randomised participants stratified by NNS naïveté^a^.

| **Variable** | **Water (*n* = 246)** | | **NNS beverages (*n* = 247)** | |
| --- | --- | --- | --- | --- |
|  | **Naïve** | **Non-naïve** | **Naïve** | **Non-naïve** |
| Participants, *n* | 60 | 186 | 59 | 188 |
| Age, y | 45.4 ±13.4 | 46.2 ±10.4 | 46.4 ±11.9 | 44.2 ±12.0 |
| Female sex, *n* (%) | 32 (53.3) | 133 (71.5) | 40 (67.8) | 140 (74.5) |
| BMI^b^, kg/m^2^ | 30.9 ±2.3 | 31.5 ±2.3 | 31.3 ±2.1 | 31.3 ±2.3 |
| Body weight, kg | 90.9 ±12.2 | 90.2 ±10.8 | 89.9 ±10.4 | 89.9 ±11.4 |

*NNS* non-nutritive sweetened.

Data are mean ±SD or *n* (%).

^a^Naïve was defined as NNS beverages comprising 0–≤25% of drink choices in the 5 y to screening; these individuals could be regular consumers of water or sugar-sweetened beverages. Non-naïve was defined as NNS beverages comprising >26–100% of drink choices in the 5 y to screening.

^b^BMI was measured at screening as part of trial eligibility assessments.

Supplemental Table 3 Baseline characteristics for all randomised participants who provided blood samples.

| **Variable** | **Water (*n* = 166)** | **NNS beverages (*n* = 165)** |
| --- | --- | --- |
| Age, y | 47.2 ±10.9 | 46.4 ±11.5 |
| Female sex, *n* (%) | 109 (65.7) | 123 (74.5) |
| BMI^a^, kg/m^2^ | 31.3 ±2.3 | 31.4 ±2.3 |
| Body weight, kg | 90.5 ±10.6 | 89.1 ±10.9 |
| NNS beverage naïveté^b^ |  |  |
| Non-naïve, *n* (%) | 122 (73.5) | 122 (73.9) |
| Naïve, *n* (%) | 44 (26.5) | 43 (26.1) |

*NNS* non-nutritive sweetened.

Data are mean ±SD or *n* (%).

^a^BMI was measured at screening as part of trial eligibility assessments.

^b^Naïve was defined as NNS beverages comprising 0–≤25% of drink choices in the 5 y to screening; these individuals could be regular consumers of water or sugar-sweetened beverages. Non-naïve was defined as NNS beverages comprising >26–100% of drink choices in the 5 y to screening.

Supplemental Table 4 Baseline characteristics for all randomised participants who were in the DXA subset.

| **Variable** | **Water (*n* = 59)** | **NNS beverages (*n* = 56)** |
| --- | --- | --- |
| Age, y | 45.6 ±11.0 | 44.3 ±12.1 |
| Female sex, *n* (%) | 38 (64.4) | 38 (67.9) |
| BMI^a^, kg/m^2^ | 30.9 ±2.2 | 31.7 ±2.0 |
| Body weight, kg | 89.6 ±10.3 | 91.5 ±12.0 |
| NNS beverage naïveté^b^ |  |  |
| Non-naïve, *n* (%) | 40 (67.8) | 39 (69.6) |
| Naïve, *n* (%) | 19 (32.2) | 17 (30.4) |

*DXA* dual-energy X-ray absorptiometry, *NNS* non-nutritive sweetened.

Data are mean ±SD or *n* (%).

^a^BMI was measured at screening as part of trial eligibility assessments.

^b^Naïve was defined as NNS beverages comprising 0–≤25% of drink choices in the 5 y to screening; these individuals could be regular consumers of water or sugar-sweetened beverages. Non-naïve was defined as NNS beverages comprising >26–100% of drink choices in the 5 y to screening.

Supplemental Table 5 Effects of different covariates on body weight at wk 52 – sensitivity analysis of complete cases data.

| **Predictor** | **B** | **SE** | **95% CI** | ***p* value** |
| --- | --- | --- | --- | --- |
| Baseline body weight | 0.951 | 0.043 | 0.867, 1.035 | < 0.001 |
| Age | –0.057 | 0.035 | –0.125, 0.011 | 0.100 |
| Male sex | –0.133 | 1.020 | –2.142, 1.877 | 0.897 |
| Self-collected body weight | 1.263 | 0.741 | –0.197, 2.723 | 0.090 |
| Non–NNS-naïve | –0.349 | 0.838 | –1.999, 1.300 | 0.677 |
| Assigned NNS beverages | –1.499 | 0.725 | –2.927, –0.072 | 0.040 |
| R^2^ adjusted | 0.776 |  |  |  |

*ANCOVA* analysis of covariance, *B* unstandardised regression coefficient, *NNS* non-nutritive sweetened.

Sensitivity analysis of the complete cases data set, which included all participants with data at baseline and wk 52. An ANCOVA was used to predict body weight at wk 52, with blinded trial group as a predictor and baseline weight, age, sex, location of weight measurement (self-collected vs. clinic-collected) and NNS beverage naïveté (non-naïve vs. naïve) as a covariate.

Supplemental Table 6 Effects of different covariates on body weight at wk 52 – primary and sensitivity analyses of multiple imputation data.

| **Predictor** | **B** | **SE** | **95% CI** | ***p* value** |
| --- | --- | --- | --- | --- |
| **Primary analysis** |  |  |  |  |
| Baseline body weight | 0.940 | 0.025 | 0.892, 0.989 | < 0.001 |
| Assigned NNS beverages | –0.850 | 0.548 | –1.927, 0.226 | 0.121 |
| R^2^ adjusted | 0.748 |  |  |  |
| **Sensitivity analysis** |  |  |  |  |
| Baseline body weight | 0.933 | 0.031 | 0.872, 0.995 | < 0.001 |
| Age | –0.089 | 0.024 | –0.135, –0.043 | < 0.001 |
| Male sex | –0.279 | 0.759 | –1.770, 1.211 | 0.713 |
| Self-collected body weight | 0.641 | 0.563 | –0.465, 1.748 | 0.255 |
| Non–NNS-naïve | –0.191 | 0.643 | –1.455, 1.072 | 0.766 |
| Assigned NNS beverages | –0.978 | 0.544 | –2.046, –0.090 | 0.073 |
| R^2^ adjusted | 0.754 |  |  |  |

*ANCOVA* analysis of covariance, *B* unstandardised regression coefficient, *NNS* non-nutritive sweetened.

Primary and sensitivity analyses of the multiple imputation data set, for which missing data were imputed using predictive mean matching (50 imputations). For the primary analysis, an ANCOVA was used to predict body weight at wk 52, with blinded trial group as a predictor and baseline weight as a covariate. For the sensitivity analysis, an ANCOVA was used to predict body weight at wk 52, with blinded trial group as a predictor and baseline weight, age, sex, location of weight measurement (self-collected vs. clinic-collected) and NNS beverage naïveté (non-naïve vs. naïve) as a covariate.

Supplemental Table 7 Effects of different covariates on body weight at wk 52 – primary and sensitivity analyses of last observation carried forward data.

| **Predictor** | **B** | **SE** | **95% CI** | ***p* value** |
| --- | --- | --- | --- | --- |
| **Primary analysis** |  |  |  |  |
| Baseline body weight | 0.958 | 0.021 | 0.916, 0.999 | < 0.001 |
| Assigned NNS beverages | –0.667 | 0.468 | –1.586, 0.253 | 0.155 |
| R^2^ adjusted | 0.808 |  |  |  |
| **Sensitivity analysis** |  |  |  |  |
| Baseline body weight | 0.951 | 0.043 | 0.867, 1.035 | < 0.001 |
| Age | –0.057 | 0.035 | –0.125, 0.011 | 0.100 |
| Male sex | –0.123 | 1.020 | –2.132, 1.886 | 0.904 |
| Self-collected body weight | 1.269 | 0.741 | –0.191, 2.728 | 0.088 |
| Non–NNS-naïve | –0.338 | 0.838 | –1.988, 1.312 | 0.687 |
| Assigned NNS beverages | –1.504 | 0.725 | –2.932, –0.077 | 0.039 |
| R^2^ adjusted | 0.776 |  |  |  |

*ANCOVA* analysis of covariance, *B* unstandardised regression coefficient, *NNS* non-nutritive sweetened.

Primary and sensitivity analyses of the last observation carried forward data set, for which missing data were imputed using participants’ last observed value. For the primary analysis, an ANCOVA was used to predict body weight at wk 52, with blinded trial group as a predictor and baseline weight as a covariate. For the sensitivity analysis, an ANCOVA was used to predict body weight at wk 52, with blinded trial group as a predictor and baseline weight, age, sex, location of weight measurement (self-collected vs. clinic-collected) and NNS beverage naïveté (non-naïve vs. naïve) as a covariate.

Supplemental Table 8 Effects of different covariates on waist circumference at wk 52 – primary and sensitivity analyses of complete cases data.

| **Predictor** | **B** | **SE** | **95% CI** | ***p* value** |
| --- | --- | --- | --- | --- |
| **Primary analysis** |  |  |  |  |
| Baseline waist circumference | 0.774 | 0.049 | 0.679, 0.870 | < 0.001 |
| Assigned NNS beverages | –1.467 | 0.851 | –3.142, 0.208 | 0.086 |
| R^2^ adjusted | 0.499 |  |  |  |
| **Sensitivity analysis** |  |  |  |  |
| Baseline waist circumference | 0.723 | 0.055 | 0.614, 0.832 | < 0.001 |
| Age | –0.010 | 0.041 | –0.089, 0.070 | 0.814 |
| Male sex | 2.023 | 1.034 | –0.013, 4.060 | 0.052 |
| Self-collected waist circumference | 0.652 | 0.894 | –1.110, 2.413 | 0.467 |
| Non–NNS-naïve | –0.072 | 0.990 | –2.021, 1.877 | 0.942 |
| Assigned NNS beverages | –1.307 | 0.858 | –2.995, 0.382 | 0.129 |
| R^2^ adjusted | 0.500 |  |  |  |

*ANCOVA* analysis of covariance, *B* unstandardised regression coefficient, *NNS* non-nutritive sweetened.

Primary and sensitivity analyses of the complete cases data set, which included all participants with data at baseline and wk 52. For the primary analysis, an ANCOVA was used to predict waist circumference at wk 52, with blinded trial group as a predictor and baseline waist circumference as a covariate. For the sensitivity analysis, an ANCOVA was used to predict waist circumference at wk 52, with blinded trial group as a predictor and baseline waist circumference, age, sex, location of circumference measurement (self-collected vs. clinic-collected) and NNS beverage naïveté (non-naïve vs. naïve) as a covariate.

Supplemental Table 9 Effects of different covariates on hip circumference at wk 52 – primary and sensitivity analyses of complete cases data.

| **Predictor** | **B** | **SE** | **95% CI** | ***p* value** |
| --- | --- | --- | --- | --- |
| **Primary analysis** |  |  |  |  |
| Baseline hip circumference | 0.781 | 0.050 | 0.683, 0.878 | < 0.001 |
| Assigned NNS beverages | –1.412 | 0.664 | –2.719, –0.104 | 0.035 |
| R^2^ adjusted | 0.494 |  |  |  |
| **Sensitivity analysis** |  |  |  |  |
| Baseline hip circumference | 0.778 | 0.052 | 0.675, 0.881 | < 0.001 |
| Age | –0.058 | 0.031 | –0.120, 0.003 | 0.063 |
| Male sex | 0.490 | 0.750 | –0.987, 1.968 | 0.514 |
| Self-collected hip circumference | 0.303 | 0.751 | –1.078, 1.684 | 0.666 |
| Non–NNS-naïve | –0.095 | 0.772 | –1.616, 1.425 | 0.902 |
| Assigned NNS beverages | –1.463 | 0.667 | –2.777, –0.150 | 0.029 |
| R^2^ adjusted | 0.494 |  |  |  |

*ANCOVA* analysis of covariance, *B* unstandardised regression coefficient, *NNS* non-nutritive sweetened.

Primary and sensitivity analyses of the complete cases data set, which included all participants with data at baseline and wk 52. For the primary analysis, an ANCOVA was used to predict hip circumference at wk 52, with blinded trial group as a predictor and baseline hip circumference as a covariate. For the sensitivity analysis, an ANCOVA was used to predict hip circumference at wk 52, with blinded trial group as a predictor and baseline hip circumference, age, sex, location of circumference measurement (self-collected vs. clinic-collected) and NNS beverage naïveté (non-naïve vs. naïve) as a covariate.

Supplemental Fig. 1 CONSORT flow diagram


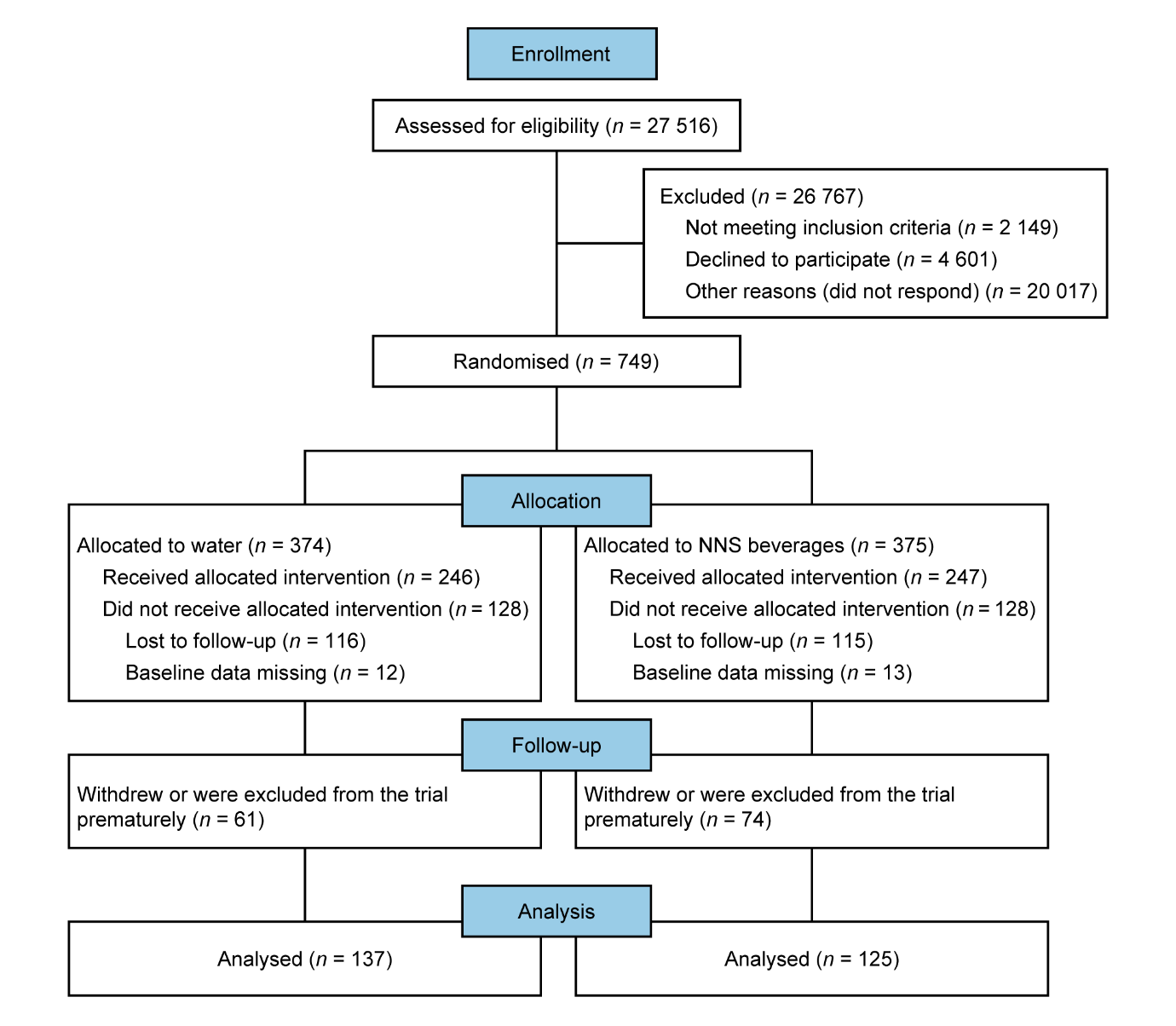


Supplemental Fig. 2 Comparison of body weight by location of weight measurement.


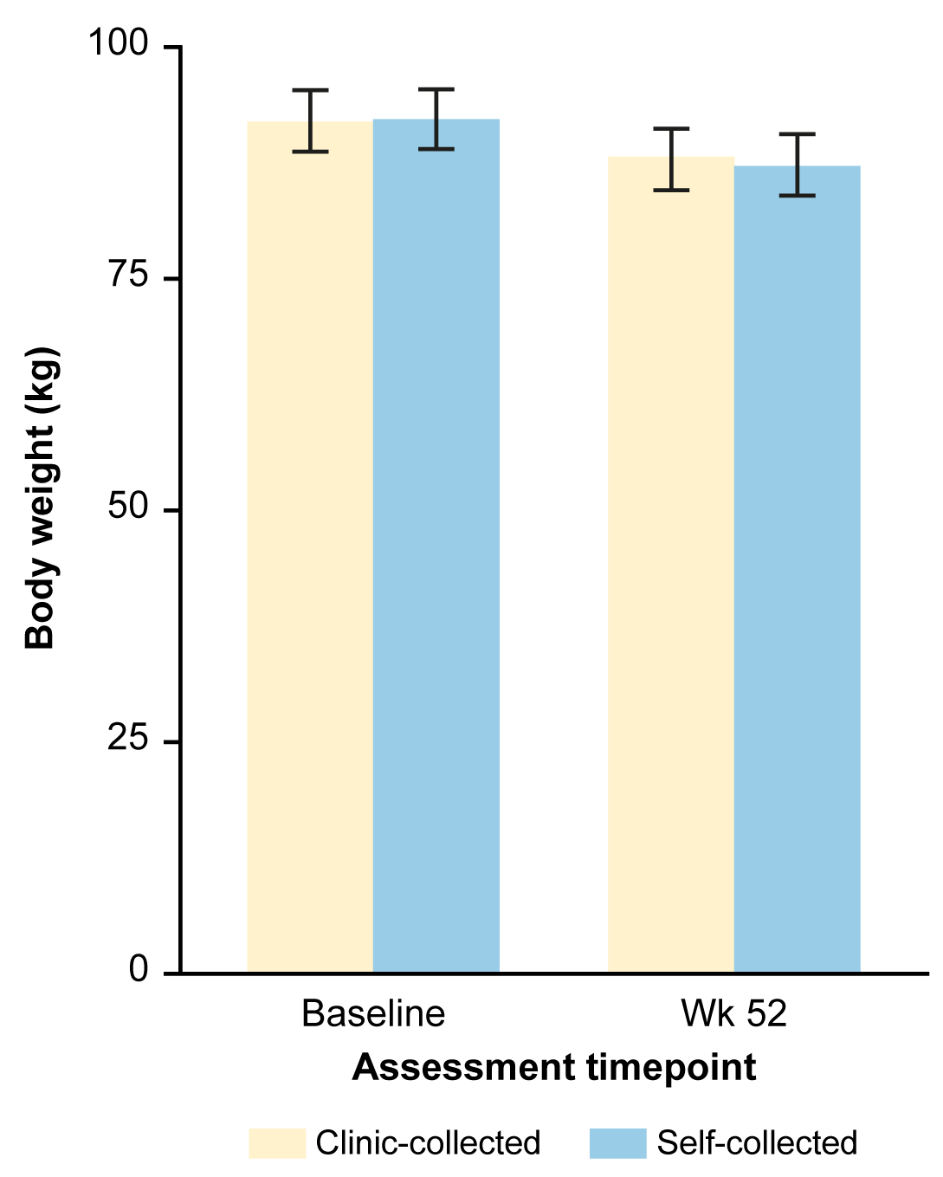


Primary analysis of the complete cases data set, which included all participants with data at baseline and wk 52. The error bars are 95% CIs.

# Supplemental references

1. Harrold JA, Hill S, Radu C, Thomas P, Thorp P, Hardman CA, et al. Effects of non-nutritive sweetened beverages versus water after a 12-week weight loss program: a randomized controlled trial. Obesity (Silver Spring). 2023;31(8):1996–2008.
